# Association of Plasma and Cerebrospinal Fluid Alzheimer Disease Biomarkers With Race and the Role of Genetic Ancestry, Vascular Comorbidities, and Neighborhood Factors

**DOI:** 10.1001/jamanetworkopen.2022.35068

**Published:** 2022-10-06

**Authors:** Ihab Hajjar, Zhiyi Yang, Maureen Okafor, Chang Liu, Teresa Waligorska, Felicia C. Goldstein, Leslie M. Shaw

**Affiliations:** 1 Department of Neurology, Emory University School of Medicine, Atlanta, Georgia; 2 Department of Neurology, University of Texas Southwestern, Dallas; 3 Department of Biostatistics and Bioinformatics, Emory University Rollins School of Public Health, Atlanta, Georgia; 4 Department of Pathology and Laboratory Medicine, University of Pennsylvania, Philadelphia; 5 Center for Neurodegenerative Disease Research, University of Pennsylvania, Philadelphia

## Abstract

**Importance:**

Differences in cerebrospinal fluid (CSF) tau Alzheimer dementia (AD) biomarkers by self-identified race have been observed in prior studies. More recently, plasma biomarkers have been gaining recognition, but whether they exhibit similar differences is unclear. Furthermore, the underlying explanation for these differences in AD biomarkers is still unexplored.

**Objectives:**

To investigate differences in plasma biomarkers by race and genetic ancestry and explore potential underlying explanations for these differences.

**Design, Setting, and Participants:**

This cross-sectional study used participant data from the Brain, Stress, Hypertension, and Aging Research Program (B-SHARP), an observational study conducted in the greater Atlanta metropolitan area. Participants were enrolled from March 1, 2016, to January 1, 2020.

**Main Outcomes and Measures:**

Main outcomes were plasma and CSF amyloid-β (Aβ) 42, Aβ40, phosphorylated tau_181_ (p-tau_181_), and neurofilament light. General linear models were used for key comparisons.

**Exposures:**

Main independent variables were self-identified race and genetic ancestry. Additional variables were cardiovascular factors, *APOE4*, educational attainment, Area Deprivation Index, and C-reactive protein (reflecting systemic inflammation state).

**Results:**

This analysis included 617 participants (mean [SD] age, 66 [7.9] years; 300 [49%] African American and 317 [51%] White; 429 [70%] with mild cognitive impairment). On the basis of self-reported race, plasma levels of Aβ42 (adjusted mean difference, −1.20 pg/mL; 95% CI, −2.33 to −0.07 pg/mL), Aβ40 (adjusted mean difference, −37.78 pg/mL; 95% CI, −60.16 to −15.39 pg/mL), p-tau_181_ (adjusted mean difference, −4.66 pg/mL; 95% CI, −7.05 to −1.90 pg/mL), and neurofilament light (adjusted mean difference, −1.58; 95% CI, −2.83 to −0.19 pg/mL) were consistently lower in African American individuals after adjusting for demographic characteristics, educational attainment, cognition, *APOE4*, and cardiovascular factors. A similar pattern was observed in the CSF biomarkers except for Aβ42 and Aβ40. Although unadjusted analyses revealed an association between these biomarkers and African ancestry, these associations were not significant after adjusting for the same covariates. Differences by self-reported race were not explained by varied cardiovascular risk factors, C-reactive protein, educational attainment, or Area Deprivation Index.

**Conclusions and Relevance:**

In this cross-sectional study of plasma biomarkers by race and genetic ancestry, the results indicated that plasma p-tau_181_, Aβ40, and NFL were lower in African American individuals based on self-reported race but not genetic ancestry. These differences were not explained by cardiovascular risks or clinical stage differences. These racial differences should be considered in clinical interpretations and clinical trial screenings to avoid an additional increase in underrepresentation of African American individuals in AD trials.

## Key Points

**Question:**

Are there differences in emerging plasma Alzheimer dementia (AD) biomarkers, as observed in cerebrospinal fluid measures, by self-reported race or genetic ancestry?

**Findings:**

In this cross-sectional study of 617 individuals with normal cognition or mild cognitive impairment, levels of plasma phosphorylated tau_181_, amyloid-β 40, and amyloid-β 42 were lower in African American individuals compared with White individuals independent of cognitive function, vascular comorbidity, and *APOE4* status. These differences were associated with self-reported race but not genetic ancestry.

**Meaning:**

Clinical and research screening activities that rely on AD biomarkers need to consider these racial differences to avoid further disparity in African American representation in AD trials.

## Teaser

This cross-sectional study of African American and White individuals examines differences in plasma biomarkers by self-reported race and genetic ancestry and factors potentially associated with these differences.

## Introduction

The prevalence of Alzheimer dementia (AD) in African American individuals is higher than among White individuals.^1,2,3^ Cerebrospinal fluid (CSF) disease biomarkers are increasingly used in clinical and research activities in AD. However, only a few studies^4,5,6,7^ have included African American individuals with measurements of biomarkers. In the few reported studies^4,5,6,7^ that specifically examined racial differences in biomarkers, African American individuals have been consistently found to have lower measures of CSF tau and phosphorylated tau_181_ (p-tau_181_). These levels were lower even after accounting for differences in cognitive performance and hippocampal volumes.^7^

Recent evidence has suggested that plasma biomarkers, including amyloid-β (Aβ) 42, Aβ40, and p-tau_181_, may be a reliable alternative to CSF measures.^8,9^ Plasma markers, such as Aβ42, Aβ40, p-tau_181_, and neurofilament light (NFL), have had promising performances in detecting AD, and such assay development may make it possible to classify individuals based on related indicators of amyloidosis (A), tauopathy (T), and neurodegeneration (N); ie, the AT(N) categories.^10,11,12,13,14,15,16^ Whether the racial disparities reported in CSF total tau and p-tau_181_ extend to the plasma measures is unclear.

Prior research on racial disparity and AD has measured race as a self-identified group. Although self-reported race is a useful construct, it is highly affected by several geographic, cultural, and sociopolitical factors.^17,18^ Genetic ancestry may provide additional understanding of racial differences and the underlying biological constructs.^19^ To our knowledge, few studies have explored this aspect of the biomarker differences in AD.

The implications and explanations for the differences by race in AD biomarkers are still evolving. On the basis of self-report, African American individuals have differential cardiovascular diseases and risks, *APOE4* dosage and effects,^20^ and social determinants of health (SDOH). For example, in a recent study,^21^ socioeconomic status was suggested to mediate the association between self-reported race and AD biomarkers. The individual Area Deprivation Index (ADI) can provide insight into the association of SDOH factors and cardiovascular and other diseases.^22,23,24,25^ Investigating the role of ADI in AD racial disparity would offer insight into the role of the environmental SDOH on AD biomarkers. This study aimed to investigate differences in plasma biomarkers by self-reported race and genetic ancestry and explore potential underlying explanations for these differences.

## Methods

Data for this cross-sectional study were drawn from the baseline assessment of participants in the Brain, Stress, Hypertension, and Aging Research Program (B-SHARP) at Emory University. B-SHARP participants undergo cognitive assessments, neuroimaging, and lumbar punctures and are subsequently enrolled in observational studies or clinical trials based on their eligibility. This analysis included 617 participants enrolled from March 1, 2016, to January 1, 2020. The Emory University Institutional Review Board approved the protocol before recruitment. Each participant provided written informed consent. This report follows the recommended Strengthening the Reporting of Observational Studies in Epidemiology (STROBE) reporting guideline.^26^

### Participant Description

The sample included adults 50 years or older with normal cognition or mild cognitive impairment (MCI) in Atlanta, Georgia. Potential study participants were identified through a referral from the Goizueta Alzheimer Disease Research Center at Emory University or through community partnerships with grassroots health education organizations, health fairs, advertisements, and mailed announcements. An appropriate study informant, defined as an individual who has regular contact with the participant at least once a week (in person or via telephone), was also identified. Participants were excluded if they had a history of stroke in the past 3 years, did not have a study informant, had a clinical diagnosis of dementia of any type, or had abnormal levels of serum thyrotropin or vitamin B_12_.

### Cognitive Assessment

Categorization of MCI and normal cognition was determined using a consensus diagnosis^27^ based on a clinician interview as well as cognitive assessment. Each participant’s evaluation underwent a review with the study physician and principal investigator (I.H.) and a neuropsychologist (F.C.G.). The criteria for MCI were as follows: Montreal Cognitive Assessment (MoCA) score of less than 26,^28^ subjective memory concerns, Clinical Dementia Rating (CDR) sum of boxes score of 0.5 and memory box score of 0.5,^29^ education-adjusted cutoff score on the Logical Memory task (delayed paragraph A only) of the Wechsler Memory Scale–Revised (Alzheimer Disease Neuroimaging Initiative) of less than 11 for 16 or more years of education, less than 9 for 8 to 15 years of education, and less than 6 for 7 or fewer years of education (the maximum score is 25), and preserved instrumental activities of daily living (Functional Activities Questionnaire) score of 7 or less.^30^ Normal cognition was defined as having no significant memory concerns beyond those expected for age, a MoCA score of 26 points or greater, a CDR sum of boxes score of 0 (including a 0 memory box score), and preserved Functional Activities Questionnaire score of 7 or less. Additional cognitive testing included episodic memory (Hopkins Verbal Learning Test–Revised), executive functioning (Trail Making Test), confrontation naming (Boston Naming Test), and attention (Digit Span Forward and Backward). The Wide Range Achievement Test–Fourth Edition reading subtest requires the individual to read words with atypical grapheme to phoneme relationships and is used as a proxy for quality of education.^31^ In the current study, this subtest was used to reflect educational quality.^32^

### Key Measures

Demographic characteristics (age, sex, race, and educational attainment), anthropometrics (weight and height), medical diagnosis, recent address (to derive ADI), and income levels were collected at baseline by interview. Brain magnetic resonance imaging (MRI) was also completed (3.0-T Trio MRI scanner, Siemens Medical Solutions). High-resolution, T1-weighted images were acquired using magnetization-prepared rapid gradient-echo imaging sequence (field of view, 256 × 256 mm^2^; 176 sagittal slices; isotropic voxel resolution, 1.0 mm^3^; repetition time, 2300 milliseconds; echo time, 2.89 milliseconds; inversion time, 800 milliseconds; flip angle, 8 °; and scan duration, 8 minutes 37 seconds). Quality checks included head motion detection and correction, Atlas registration confirmation, and visual inspection of images.

Hippocampal volume and other volumetric measurements were calculated using the FreeSurfer package with manual supervision. Quality checks were performed for each scan. Left and right hippocampal volumes were obtained and combined to derive the total hippocampal volume. Intracranial volume was also derived from this analysis. Volumetric measurements using FreeSurfer provide similar estimates to a fully manual procedure.^33^ We used intracranial volume–adjusted hippocampal volume to reflect the degree of neurodegeneration for each participant.^16^ Microvascular disease was reflected by white matter lesions or hyperintensities.

### Biospecimen Collection

After a fast of no less than 6 hours, CSF samples were collected via lumbar puncture using 24-G Sprotte atraumatic spinal needles. Samples were collected in sterile polypropylene tubes, separated into 0.5-mL aliquots, and stored at −80 °C. Blood was also drawn at the same visit. The CSF and plasma samples obtained at the same time were subsequently shipped and analyzed at the Biomarker Research Laboratory at the University of Pennsylvania.^34^

### Biomarker Measurements

Aβ42, Aβ40, p-tau, and total tau were quantified directly from the aliquot tube using Aβ1-42, Aβ1-40, p-tau_181_, and total tau assays. The CSF samples were tested using a fully automated chemiluminescent enzyme immunoassay (Lumipulse G1200, Fujirebio Diagnostics). Operators were trained and tested during 3 days to ensure competency with the analyzer and sample handling procedures. Quality control testing was performed beginning each test day to ensure control levels (low, medium, and high) were within target ranges. The Lumipulse Aβ1-42 assay has been standardized according to certified reference materials developed by the International Federation of Clinical Chemistry and Laboratory Medicine Working Group for CSF Proteins.

Both NFL and p-tau_181_ were measured using the Simoa Platform Version 2 Advantage Kit (Quanterix Corp) and were used in a fully automated 2-step sandwich immunoassay. In the first step, the sample is drawn by the instrument pipettor, combined with anti–p-tau_181_–coated paramagnetic capture beads and biotinylated detector antibodies in a reaction cuvette, and incubated. After the incubation, capture beads are collected with a magnet and washed. After washing, a conjugate of streptavidin-β-galactosidase is mixed with the capture beads for the second assay step, during which streptavidin-β-galactosidase binds to the biotinylated detector antibodies, resulting in enzyme labeling of captured p-tau_181_. After a second wash, the capture beads are resuspended in a resorufin β-d-galactopyranoside substrate solution. Digital processing occurs when beads are transferred to the Simoa array disk. Individual capture beads are then sealed within microwells in the array. If p-tau_181_ has been captured and labeled, the β-galactosidase hydrolyzes the resorufin β-d-galactopyranoside substrate into a fluorescent product that provides the signal for measurement. Primary reference standards are recombinant human p-tau_181_in phosphate buffer. No international reference material is available for p-tau_181_. Recombinant human p-tau_181_ was chosen for standardization over other candidate p-tau_181_ materials (eg, synthetic p-tau_181_ peptide) on the basis of availability and assay performance. Calibrators are stored frozen and thawed at the point of use. Similar steps are followed for NFL quantification.

To compare our results with previously published reports, we also provide results of the CSF AD biomarkers based on the now discontinued multiplex xMAP Luminex platform (Luminex Corp) with Innogenetics (INNO-BIA AlzBio3; for research use–only reagents) immunoassay kit–based reagents. Prior racial comparisons have used this platform.^4,6,7^

### Genetic Testing

DNA was extracted from buffy coat using the GenePure kit (Qiagen) following the manufacturer's recommended protocol. The genome-wide association study was completed using the GeneChip Axiom Precision Medicine Research Array Affymetrix). We calculated individual African ancestry proportions for our study participants using the Admixture software^35^ with 5-fold cross-validation, coupled with individuals from HapMap phase 3 reference data that represented African ancestry in the Southwest US, Utah residents with Northern and Western European ancestry from the Centre d'Etude du Polymorphism Humain collection, Yoruba in Ibadan, Nigeria, Japanese in Tokyo, Japan, and Han Chinese in Beijing, China ancestral populations. We included the genetic ancestry as the percentage of African ancestry in this analysis. Individuals were also genotyped for *APOE* using genotyping from the GeneChip Axiom Precision Medicine Research Array or using TaqMan assays (ThermoFisher Scientific) to determine the genotype for rs7412 and rs429358 that define the *APOE* haplotype.

### Statistical Analysis

All statistical analyses and data processing were conducted using R software, version 4.0.5 (R Foundation for Statistical Computing) and SAS software, version 9.4 T1M6 (SAS Institute Inc). A 2-sided *P* < .05 was considered statistically significant. Study participants’ characteristics were compared between the 2 racial groups (African American vs White) using the *t* test or χ^2^ test and presented as means (SDs) or numbers (percentages). All data distributions were checked for normality, and those deviating from normality were log transformed.

Associations between biomarkers (CSF or plasma Aβ40, Aβ42, total tau, p-tau_181_, and NFL) and self-reported race or genetic (African) ancestry were investigated using general linear models. Multivariable analyses were adjusted for age, sex, educational attainment, MoCA score (reflecting disease severity), *APOE4*, hypertension, diabetes, and serum creatinine level. Our selection of covariates was based on the statistically significant differences between the 2 groups and to identify comorbid-independent associations. For the race comparisons, we present unadjusted means and covariate-adjusted least-square means as well as covariate-adjusted mean differences (MDs). Genetic ancestry was analyzed as a continuous variable, and we present unadjusted and covariate-adjusted slopes for the associations between ancestry and biomarkers. To test possible underlying explanatory factors, we compared adjusted and unadjusted test results and conducted formal mediation analyses using structural equation modeling with mediation to conduct selected mediation analyses.^36^ The mediation procedure estimates mediation effects from observational data to assess whether mean sitting systolic blood pressure, global inflammation (C-reactive protein), body mass index (BMI, calculated as weight in kilograms divided by height in meters squared), *APOE4*, and ADI mediated the association between self-reported race and biomarkers. Age, sex, and years of education were set as potential confounders.

## Results

### Sample Description by Self-reported Race

This analysis included 617 participants (mean [SD] age, 66 [7.9] years; 300 [49%] African American and 317 [51%] White; 429 [70%] with MCI). Characteristics of the 2 comparison groups based on self-reported race are provided in Table 1. The African American group was younger (mean [SD] age, 63.60 [6.83] years) than the White group (mean [SD] age, 67.40 [8.48] years; *P* < .001) and had a higher prevalence of cardiovascular risks (hypertension [230 (80.7%) vs 140 (48.6%)], diabetes [77 (27.0%) vs 30 (10.4%)], and higher mean [SD] BMI [31.64 (7.26) vs 27.52 (5.98)], *P* < .001 for all) and MCI (220 [74.1%] vs 209 [66.1%], *P* = .04). African American individuals also had higher mean (SD) ADI scores (5.43 [1.88] vs 3.22 [1.92], *P* < .001), lower mean (SD) educational attainment (14.62 [2.53] vs 16.18 [2.58] years, *P* < .001), and lower mean (SD) WRAT scores (97.10 [16.74] vs 113.38 [13.72], *P* < .001). African American individuals had lower performances on the Hopkins Verbal Learning Test delayed recall (mean [SD] scores, 7.12 [3.02] vs 7.69 [3.72]; *P* = .04) and the Trail Making Test part A (mean [SD] scores, 41.26 [16.59] vs 37.29 [17.61]; *P* = .006) and part B (mean [SD] scores, 144.69 [80.52] vs 111.51 [75.64]; *P* < .001), among other tests (Table 1). However, both groups had similar performance on Logical Memory story recall, CDR scores, hippocampal volumes, and microvascular disease burden (white matter hyperintensities). They also had equivalent *APOE4* distribution.

**Table 1.** Comparison of Key Characteristics of White and African American Study Participants^a^

Characteristic	African American participants (n = 300)	White participants (n = 317)	*P* value
**Demographic characteristics**			
Age, mean (SD), y	63.60 (6.83)	67.40 (8.48)	<.001
Sex			
Female	194 (64.7)	183 (57.7)	.08
Male	106 (35.3)	134 (42.3)	
Social determinants			
Educational attainment, mean (SD), y	14.62 (2.53)	16.18 (2.58)	<.001
Salary level			
<25 000	128 (57.1)	100 (40.2)	<.001
25 000-50 000	60 (26.8)	55 (22.1)	
50 000-80 000	26 (11.6)	40 (16.1)	
>80 000	10 (4.5)	54 (21.7)	
ADI score, mean (SD)	5.43 (1.9)	3.22 (1.9)	<.001
**Clinical characteristics**			
Hypertension			
No	55 (19.3)	148 (51.4)	<.001
Yes	230 (80.7)	140 (48.6)	
Diabetes			
No	208 (73.0)	257 (89.6)	<.001
Yes	77 (27.0)	30 (10.4)	
GFR, mean (SD), mL/min/1.73 m^2^	85.57 (18.92)	77.24 (14.36)	<.001
CRP, mean (SD), mg/dL	0.54 (0.66)	0.43 (1.64)	.38
BMI, mean (SD)	31.64 (7.26)	27.52 (5.98)	<.001
**Cognitive characteristics**			
Consensus diagnosis			
MCI	220 (74.1)	209 (66.1)	.04
Normal	77 (25.9)	107 (33.9)	
Test scores			
MoCA, mean (SD)	21.77 (3.82)	23.99 (3.58)	<.001
Logical Memory, mean (SD)			
Story score 1	12.08 (4.07)	12.05 (4.85)	.94
Story score 2	10.44 (4.20)	10.32 (5.94)	.80
CDR, total score			
0	88.00 (47.06)	113.00 (43.97)	.56
0.5	99.00 (52.94)	144.00 (56.03)	
HVLT-R delayed recall, mean (SD)	7.12 (3.02)	7.69 (3.72)	.04
Trail Making Test, mean (SD)			
Part A test completion time	41.26 (16.59)	37.29 (17.61)	.006
Part B test completion time	144.69 (80.52)	111.51 (75.64)	<.001
Boston Naming Test total correct	13.18 (1.70)	14.17 (1.22)	<.001
Digit span forward score (maximum span)	9.04 (2.13)	9.76 (2.27)	<.001
Digit span backward score (maximum span)	5.35 (2.17)	6.20 (2.55)	<.001
WRAT-4 total correct score	97.10 (16.74)	113.38 (13.72)	<.001
Neuroimaging			
Hippocampal volume, mean (SD), mm^3^	7246.60 (847.94)	7260.30 (1147.60)	.89
WML volume, mean (SD), mm^3^	3570.40 (5749.20)	3950.40 (5902.90)	.50
**Genetic characteristics**			
*APOE4*			
E4	112 (42.1)	121 (43.5)	.79
Non-E4	154 (57.9)	157 (56.5)	
Genetic ancestry, mean (SD)			
East Asian	0.01 (0.04)	0.01 (0.03)	.20
African	0.79 (0.11)	0.01 (0.02)	<.001
European	0.19 (0.10)	0.98 (0.04)	<.001

Abbreviations: ADI, Area Deprivation Index; BMI, body mass index (calculated as weight in kilograms divided by height in meters squared); CDR, Clinical Dementia Rating; CRP, C-reactive protein; GFR, glomerular filtration rate; HVLT-R, Hopkins Verbal Learning Test–Revised; MCI, mild cognitive impairment; MoCA, Montreal Cognitive Assessment; WML, white matter lesion; WRAT-4, Wide Range Achievement Test–Fourth Edition.

SI conversion factors: To convert CRP to milligrams per liter, multiply by 10.

^a^ Data are presented as number (percentage) of study participants unless otherwise indicated.

### Plasma AD Biomarker Differences by Self-reported Race

Compared with White participants, African American participants had lower levels of plasma p-tau_181_ (adjusted MD, −4.66 pg/mL; 95% CI, −7.05 to −1.90 pg/mL) and NFL (MD, −1.58 pg/mL; 95% CI, −2.83 to −0.19 pg/mL). Furthermore, although the Aβ42 (MD, −1.20 pg/mL; 95% CI, −2.33 to −0.07) and Aβ40 (MD, −37.78 pg/mL; 95% CI, −60.16 to −15.39) levels were lower in African American participants, differences in Aβ40/Aβ42 ratio MD were not statistically significant (MD, 0.01; 95% CI, 0-0.01; *P* = .08). These results are given in Table 2.

**Table 2.** Unadjusted Mean and Covariate-Adjusted LSM Concentrations of Plasma and CSF Alzheimer Dementia Biomarkers and Ratios by Race

Biomarker	Unadjusted mean (SD)			Adjusted LSM (SE)^a^			Adjusted mean difference (95% CI)
	African American participants	White participants	*P* value	African American participants	White participants	*P* value	
Plasma							
Aβ42, pg/mL	10.35 (3.43)	9.12 (3.47)	.02	8.43 (0.47)	9.62 (0.39)	.04	−1.20 (−2.33 to −0.07)
Aβ40, pg/mL	160.68 (50.74)	186.79 (59.75)	.002	147.30 (9.28)	185.08 (7.67)	.001	−37.78 (−60.16 to −15.39)
p-tau_181_, pg/mL^b^	17.99 (7.54)	21.78 (9.59)	.002	18.05 (1.05)	22.70 (1.20)	.004	−4.66 (−7.05 to −1.90)
Aβ42/Aβ40	0.07 (0.02)	0.05 (0.02)	<.001	0.06 (0.00)	0.05 (0.00)	.08	0.01 (0 to 0.01)
NFL, pg/mL^b^	11.19 (6.38)	13.41 (6.18)	<.001	12.06 (0.52)	13.64 (0.57)	.03	−1.58 (−2.83 to −0.19)
CSF							
AlzBio Innotest							
Aβ42, pg/mL	278.71 (99.26)	260.46 (95.91)	.08	272.51 (10.26)	255.53 (9.24)	.15	16.97 (−6.26 to 40.21)
Total tau, pg/mL^b^	42.61 (20.24)	60.67 (31.49)	<.001	44.80 (2.52)	61.27 (3.11)	<.001	−16.46 (−21.82 to −10.37)
p-tau_181_, pg/mL^b^	14.05 (6.86)	18.46 (10.40)	<.001	14.38 (0.82)	18.19 (0.93)	<.001	−3.81 (−5.56 to −1.83)
Lumipulse							
Aβ42, pg/mL	740.70 (370.23)	634.98 (262.92)	.21	808.87 (138.3)	680.46 (138.9)	.11	128.41 (−31.07 to 287.89)
Aβ40, pg/mL	9584.5 (3358.1)	11439 (3382.9)	.02	8688.9 (2059)	10231 (2068)	.20	−1541.64 (−3916.31 to 833.04)
Total tau, pg/mL^b^	267.21 (143.52)	454.55 (269.61)	<.001	250.25 (76.79)	436.82 (134.6)	.003	−186.57 (−261.17 to −80.29)
p-tau_181_, pg/mL^b^	37.67 (21.77)	64.81 (44.15)	<.001	30.97 (9.88)	55.84 (17.89)	.002	−24.87 (−34.41 to −11.09)
Aβ42/Aβ40	0.08 (0.03)	0.06 (0.02)	<.001	0.09 (0.01)	0.07 (0.01)	.002	0.02 (0.01 to 0.03)
Simoa							
NFL, pg/mL^b^	740.11 (342.20)	902.23 (374.76)	<.001	824.64 (35.97)	918.64 (35.80)	.034	−94 (−174.14 to −5.23)

Abbreviations: Aβ, amyloid β; CSF, cerebrospinal fluid; LSM, least-square mean; NFL, neurofilament light; p-tau, phosphorylated tau.

^a^ Derived from the general linear models after adjustment for age, sex, educational attainment, Montreal Cognitive Assessment score, *APOE4*, hypertension, diabetes, and creatinine level.

^b^ Derived from the logarithmic transformation of variables.

### CSF Biomarker Differences by Self-reported Race

As indicated in Table 2, the levels of total tau and p-tau_181_ were consistently lower in the CSF of African American participants on the historical platform (Luminex Innotest) and on the more recently available platform (Lumipulse). These differences remained significant after adjusting for age, sex, educational attainment, MoCA scores, and *APOE4* status. They also did not alter after adjusting for the presence of hypertension, diabetes, and kidney function. On the basis of the Lumipulse platform analytical results, the adjusted MDs were −186.57 pg/mL (95% CI, −261.17 to −80.29 pg/mL) in total tau and −24.87 pg/mL (95% CI, −34.41 to −11.09 pg/mL) in p-tau_181_. Although no differences were found between Aβ42 and Aβ40, the Aβ42/Aβ40 ratio was higher in African American participants (MD, 0.02; 95% CI, 0.01 to 0.03).

### Biomarker Differences by Genetic Ancestry

White participants had a 98% predominance of European ancestry, whereas African American participants had 79% African ancestry and 19% European ancestry. Both had 0.01% Asian ancestry. Before adjusting for potential confounders, the percentage of African ancestry had a similar association with AD biomarkers as noted in self-reported race. We report estimates with 95% CIs and have found that a higher level of genetic African ancestry was associated with lower CSF total tau (estimate, −25.07; 95% CI, −40.24 to −9.90; *P* = .001) and p-tau_181_ in CSF (estimate, −95.67; 95% CI, −184.93 to −6.42; *P* = .04) and plasma (estimate, −7.26; 95% CI, −13.2 to −1.32; *P* = .02) and a higher Aβ42/Aβ40 ratio in CSF (estimate, 0.05; 95% CI, 0.02-0.08; *P* = .003) and plasma (estimate, 0.01; 95% CI, 0-0.02; *P* = .009). However, in contrast to self-reported race, adjusting for potential confounders rendered these associations nonsignificant. These results are given in Table 3.

**Table 3.** Associations Between African Ancestry and Alzheimer Dementia Biomarkers

Biomarker	Unadjusted		Adjusted^a^	
	Estimate (SE)^b^	*P* value	Estimate (SE)^b^	*P* value
Plasma				
Aβ42	0.55 (0.99)	.58	−3.11 (3.77)	.41
Aβ40	−35.40 (13.82)	.01	−96.97 (65.27)	.14
p-tau_181_	−7.26 (3.00)	.02	10.36 (16.15)	.52
Aβ42/Aβ40	0.01 (0.01)	.009	0.02 (0.02)	.40
NFL	−3.97 (1.13)	<.001	−0.26 (5.69)	.96
CSF				
AlzBio Innotest				
Aβ42	23.63 (14.10)	.10	−12.38 (86.19)	.89
Total tau	−25.07 (7.69)	.001	−83.27 (46.67)	.08
p-tau_181_	−8.79 (2.04)	<.001	−11.85 (11.85)	.32
Lumipulse				
Aβ42	133.21 (113.5)	.25	157.52 (3186)	.96
Aβ40	−3453 (1886)	.08	−17393 (49 774)	.73
Total tau	−575.4 (253.1)	.03	1331.4 (6200)	.83
p-tau_181_	−95.67 (43.42)	.04	110.53 (1096)	.92
Aβ42/Aβ40	0.05 (0.02)	.003	0.18 (0.41)	.67
Simoa				
NFL	−322.5 (93.65)	<.001	−757.2 (499.9)	.13

Abbreviations: Aβ, amyloid β; CSF, cerebrospinal fluid; NFL, neurofilament light; p-tau, phosphorylated tau.

^a^ Adjustment for age, sex, education years, Montreal Cognitive Assessment score, *APOE4*, hypertension, diabetes, and creatinine.

^b^ Estimates are the slope of the association between biomarker level and percentage with African ancestry.

### Analysis by Cognitive and *APOE4* Status

The overall trends noted in the full sample were consistent in the normal cognition and MCI subgroups except for the CSF and plasma p-tau_181_ and NFL, where the CSF p-tau_181_ differences were only significant in the MCI group (15.7 in African American individuals vs 23.4 in White individuals, *P* = .001), whereas for plasma and CSF NFL, the differences were significant in the normal cognition group (CSF NFL: 748.1 in African American individuals vs 901.2 in White individuals, *P* = .003; plasma NFL: 10.5 in African American individuals vs 13.5 in White individuals, *P* < .001) (Figure). Biomarker differences did not alter based on *APOE4* status (eFigure in the Supplement).

**Figure.**
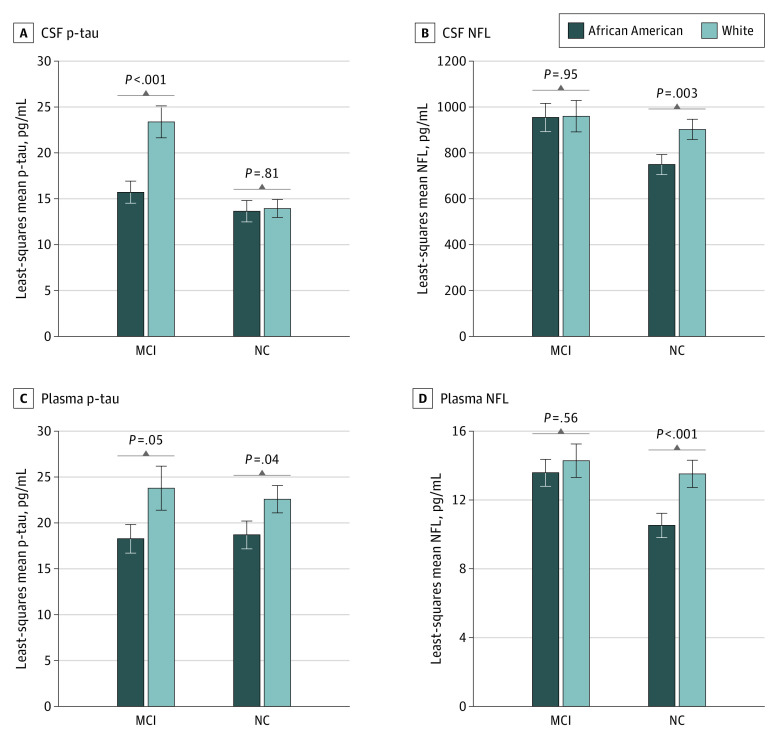
Plasma and Cerebrospinal Fluid (CSF) Biomarkers That Differ by Cognitive Status (Mild Cognitive Impairment [MCI] and Normal Cognition [NC]) Between White and African American Individuals *P* values were obtained from a model adjusted for age, sex, educational level, Montreal Cognitive Assessment score, *APOE4* status, hypertension, diabetes, and serum creatinine levels. p-tau indicates phosphorylated tau.

### Potential Explanatory Factors

In self-reported race analyses, we did not identify any potential factor that might provide an explanation by comparing unadjusted with adjusted analyses (Table 2), except for the possibility of plasma differences in the Aβ42/Aβ40 ratio, where adjustment for clinical factors rendered the difference nonsignificant (White vs African American participants: 0.07 [0.02] vs 0.05 [0.02]; *P* < .001 before adjustment and 0.06 [0.00] vs 0.05 [0.00]; *P* = .08 after adjustments; adjusted MD, 0.01; 95% CI, 0-0.01). Additional mediation analyses using structural equation modeling for the potential role of C-reactive protein, BMI, mean systolic blood pressure, *APOE4* status, and ADI revealed no mediating effects. These results are provided in the eTable in the Supplement.

## Discussion

This study suggests that the observed differences in AD biomarkers by self-reported race are also observed in plasma-based biomarkers and in CSF-based biomarkers on the Lumipulse platform. Our study suggests that African American individuals also have lower plasma p-tau_181_ and NFL levels compared with White individuals and confirms that CSF measures using the more recent Lumipulse platform have a similar pattern, as previously reported using the now discontinued Innotest.^6^ To our knowledge, this the first report to provide such evidence for both biomarkers and uses one of the largest samples of African American individuals (n = 300) included in biomarker studies.

The importance of biomarkers in non-White populations is still unclear. A recent study by Brickman et al^37^ suggests that plasma p-tau_181_ has a higher predictive performance in non-White compared with White individuals for neuropathological but not clinical AD diagnosis. Hence, it is critical to understand the differences at the group level to improve accuracy in defining AT(N) and relevant diagnostic cutoffs.

All prior racial comparisons have relied on self-reported race. Although it remains a fundamental aspect of understanding disparities in AD, consideration for genetic ancestry adds significant rigor to the field. In this study, we observed a divergence between self-reported race and genetic ancestry when comparing biomarker levels. The self-reported race comparisons were robust and did not change after adjusting the analyses for clinical and social factors included in our study, whereas the genetic ancestry differences were no longer significant after adjusting for these potential confounders. Taken together, this observation may support that the observed differences are likely associated with the social construct of race rather than an underlying genetic factor related to ancestry. The role of ancestry offers insight into a potential genetic underpinning for the phenotypic differences, and our analysis suggests that the observed biomarker differences are unlikely to be associated with the African ancestry. Additional explanations may include differences in disease classification and staging or other unmeasured factors. Additional studies into expanding potential explanatory factors beyond what was included in this study and described below might offer more insight.

One key factor that has been previously proposed as a possible explanation for the consistently lower tau measures in African American individuals is the potential for higher vascular contribution to cognitive impairment in this high-risk group. Our study does not support this explanation in multiple ways. First, accounting for the differences in vascular risk factors (hypertension, diabetes, blood pressure, BMI, and kidney function) by adjustments or mediation analyses did not explain the racial differences based on self-report. We also did not observe a difference between races in the burden of cerebral microvascular disease. Another explanation might be the differential inflammatory pathways. We did not see a role for C-reactive protein, albeit a global and nonspecific inflammatory marker. More specific measures of neuroinflammation might be more useful, and future studies will need to account for these measures.

A prior study^6^ and a recent metanalysis^38^ found significant differences for CSF tau between African American and White individuals but no consistent differences in Aβ markers measured on the Innotest platform. Our study suggests that Aβ42 and Aβ40 along with the ratio may be higher in African American individuals, but the ratio did not survive covariate adjustments in plasma. These data suggest that if the plasma ratio is to be used, adjustments for potential confounders might lower the association of race with the differences in these measures.

Levels of NFL have been found to be elevated and associated with cognitive decline and brain atrophy in individuals with AD.^39^ Our study found lower plasma and CSF NFL levels in African American individuals compared with White individuals. Neurofilament light may reflect overall neuronal injury and may be considered a biomarker for neurodegeneration. However, when considering more traditional measures of neurodegeneration, such as hippocampal volume, we did not find any significant difference by race. Hence, these differential levels are unlikely to be a reflection of earlier or different disease stages. Rather, it might be postulated that African American individuals demonstrate lower levels of many known proteins linked to AD neurodegenerative pathways, and alternative pathways may be at play. This theory is further supported by a recent report^40^ that African American individuals have lower levels of triggering receptor expressed on myeloid cells 2, which is a mediator of the immune response to amyloid plaques.

The advantage of our study lies in its relatively large proportion of African American participants (n = 300) compared with most prior studies^4,6^ in this area. In addition, the African American sample had comparable disease stages reflected by the CDR measure. Another advantage is the measurement of biomarkers on multiple platforms obtained from the same acquisition time and participants, thus providing more reliable comparisons across older and newer platforms, which revealed a consistent difference in AD biomarkers by race.

Our study has clinical and research implications for diagnostic thresholds and screening cutoffs. Relying on racially agnostic cutoffs, especially for tau markers, may be biased toward underdetection of AD or higher screen fail rates for clinical trials, further propagating underrepresentation in AD research. The lower levels of biomarkers are not necessarily indicative of lower likelihood of AD. Our study suggests that disease stages and other evidence of neurodegeneration are similar between the 2 groups, and preliminary evidence suggests a higher predictive ability of plasma p-tau_181_ in African American individuals.^37^

### Limitations

Because of the cross-sectional design of this study, the clinical relevance of differences in plasma and CSF biomarkers as a function of race awaits longitudinal investigations to examine their association with dementia risk and trajectory of cognitive decline. In addition, the measurement of social determinants of health is limited to the ADI and income level, both of which are focused on socioeconomic status. Future studies should broaden the phenotyping of social determinants to include other important contributors, such as neighborhood and physical environment (eg, housing, transportation, safety, walkability, and parks), health care (eg, coverage, availability, practitioner cultural competence, and quality of care), and the community and social context (eg, discrimination, social integration, support systems, and community engagement). Finally, apart from white matter hyperintensities, we did not have additional vascular MRI measures, such as microbleeds and infarcts, to perform a more fine-grained assessment of the association of cerebrovascular disease with biomarkers and potential racial differences.

## Conclusions

In this cross-sectional study, novel plasma AD biomarkers exhibited group-level differences by self-reported race independent of cognitive status. These differences are unlikely to be associated with underlying African ancestry and could not be explained by vascular comorbidities, global inflammatory state, *APOE4,* or other SDOH. The use of plasma biomarkers for diagnostic or research screening should account for these group-level differences based on self-identified race.

## Notes

Supplement. **eFigure.** Selected Biomarkers According to Their Apolipoprotein E (APOE) ε4 Genotype: Positive and Negative Between White and African American **eTable.** Mediation Effect Summary (P values)
