# Supplementary material for: Association of Plasma and Cerebrospinal Fluid Alzheimer Disease Biomarkers With Race and the Role of Genetic Ancestry, Vascular Comorbidities, and Neighborhood Factors
Source: JAMA Netw Open. 2022 Oct 6;5(10):e2235068. doi: 10.1001/jamanetworkopen.2022.35068 (PMC9539715; doi:10.1001/jamanetworkopen.2022.35068)
Supplement: Supplement. — eFigure. Selected Biomarkers According to Their Apolipoprotein E (APOE) ε4 Genotype: Positive and Negative Between White and African American eTable. Mediation Effect Summary (P values) [file jamanetwopen-e2235068-s001.pdf]

Supplementary Online Content

Hajjar I, Yang Z, Okafor M, et al. Association of plasma and cerebrospinal fluid Alzheimer disease biomarkers with race and the role of genetic ancestry, vascular comorbidities, and neighborhood factors. *JAMA Netw Open*. 2022;5(10):e2235068. doi:10.1001/jamanetworkopen.2022.35068

eFigure. Selected Biomarkers According to Their Apolipoprotein E (APOE) ε4 Genotype: Positive and Negative Between White and African American

eTable. Mediation Effect Summary (P Values)

This supplementary material has been provided by the authors to give readers additional information about their work.

© 2022 Hajjar I et al. *JAMA Network Open.*

eFigure. Selected Biomarkers According to Their Apolipoprotein E (APOE) ε4 Genotype: Positive and Negative Between White and African American

P-value were obtained from t-test.

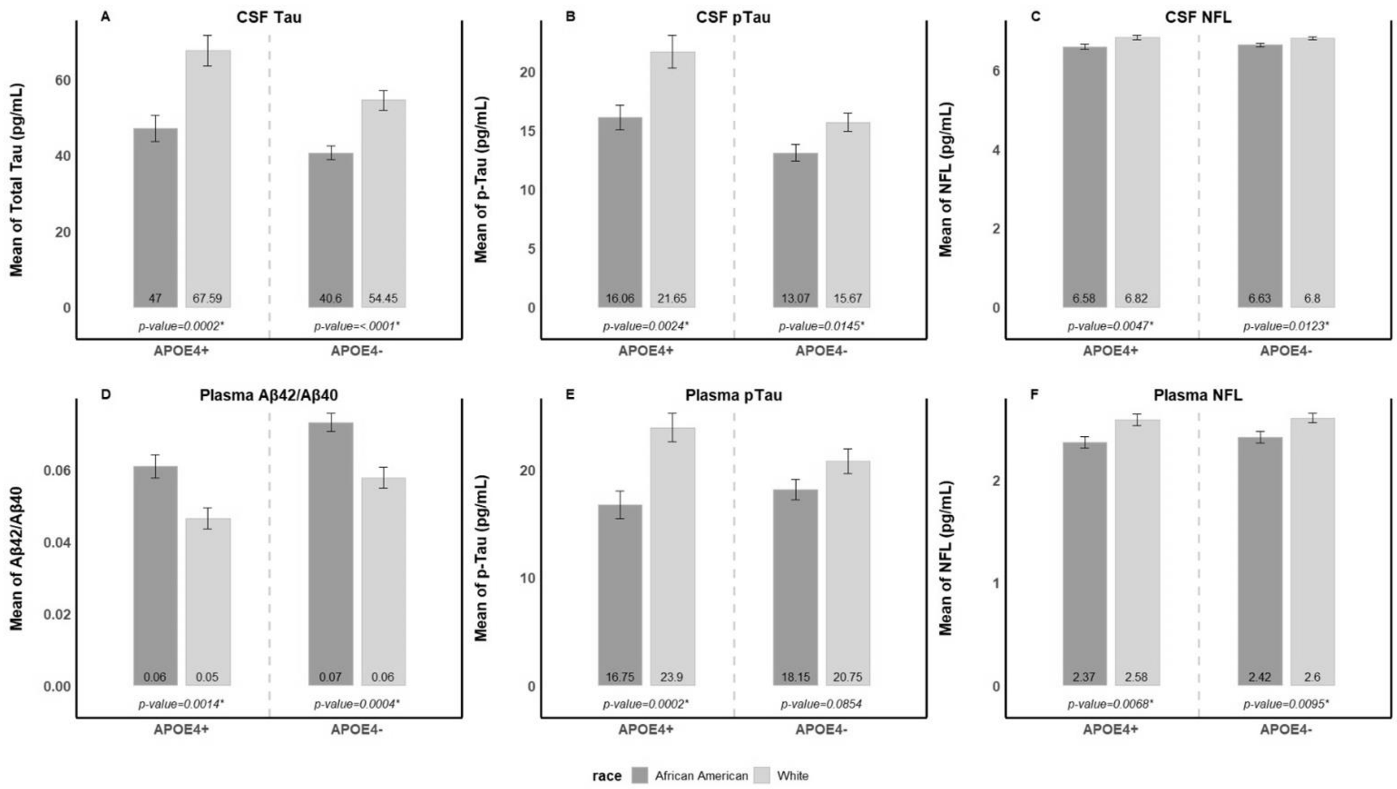

© 2022 Hajjar I et al. *JAMA Network Open.*

eTable. Mediation Effect Summary (P Values)

Mediation		M1/CRP			M1/Mean SBP Siting			M1/BMI		
Source	Variables	Total Effect	Direct Effect	Indirect Effect	Total Effect	Direct Effect	Indirect Effect	Total Effect	Direct Effect	Indirect Effect
CSF AlzBio Innotest	Aβ42	0.10	0.13	0.71	0.033	0.063	0.46	0.13	0.25	0.30
	Tau	<.0001	<.0001	0.70	<.0001	<.0001	0.67	<.0001	<.0001	0.41
	$pTau_{181}$	0.0052	0.0033	0.67	0.0036	0.0017	0.25	0.0023	0.0003	0.040
CSF LUMIPULSE	Aβ42	0.34	0.40	0.97	0.25	0.25	0.96	0.27	0.29	0.81
	Aβ40	0.14	0.22	0.77	0.14	0.15	0.96	0.19	0.12	0.81
	Tau	0.060	0.017	0.69	0.011	0.011	0.99	0.034	0.016	0.81
	$pTau_{181}$	0.041	0.031	0.74	0.024	0.024	0.97	0.028	0.017	0.82
	Aβ42/Aβ40	0.018	0.019	0.78	0.011	0.010	0.96	0.046	0.014	0.81
CSF, SIMOA	NFL	0.15	0.16	0.82	0.20	0.13	0.36	0.20	0.32	0.27
Plasma	Aβ42	0.14	0.15	0.89	0.16	0.24	0.29	0.14	0.45	0.11
	Aβ40	0.060	0.041	0.65	0.0064	0.0061	0.79	0.0050	0.0028	0.30
	$pTau_{181}$	0.019	0.022	0.98	0.019	0.075	0.15	0.023	0.057	0.26
	Aβ42/Aβ40	0.42	0.32	0.64	<.0001	<.0001	0.33	<.0001	0.0001	0.27
	NFL	0.24	0.24	0.88	0.35	0.26	0.38	0.29	0.79	0.016

Mediation		M1/ ADI			M1/APOE4		
Source	Variables	Total Effect	Direct Effect	Indirect Effect	Total Effect	Direct Effect	Indirect Effect
CSF AlzBio Innotest	Aβ42	0.11	0.74	0.10	0.15	0.17	0.50
	Tau	<.0001	0.0004	0.70	<.0001	<.0001	0.48
	$pTau_{181}$	0.0099	0.036	0.99	0.0017	0.0018	0.58
CSF LUMIPULSE	Aβ42	0.43	0.92	0.50	0.47	0.37	0.77
	Aβ40	0.11	0.064	0.30	0.089	0.072	0.78
	Tau	0.016	0.046	0.66	0.024	0.0075	0.77
	$pTau_{181}$	0.034	0.061	0.57	0.037	0.013	0.77
	Aβ42/Aβ40	0.017	0.10	0.95	0.050	0.0023	0.77
CSF, SIMOA	NFL	0.27	0.080	0.14	0.19	0.18	0.91
Plasma	Aβ42	0.25	0.48	0.87	0.16	0.12	0.54
	Aβ40	0.014	0.030	0.53	0.0062	0.0055	0.67
	$pTau_{181}$	0.023	0.021	0.43	0.020	0.019	0.70
	Aβ42/Aβ40	0.0001	0.0021	0.59	<.0001	<.0001	0.52
	NFL	0.28	0.052	0.050	0.31	0.33	0.59

© 2022 Hajjar I et al. *JAMA Network Open.*
